# Associate plant parasitic nematodes to weed species in some newly reclaimed lands

**DOI:** 10.1038/s41598-023-49357-x

**Published:** 2023-12-11

**Authors:** Ghena Mamdouh AbdelRazek, Mohamed Abdelaziz Balah

**Affiliations:** Plant Protection Department, Desert Research Center, El-Mataria, Cairo, Egypt

**Keywords:** Ecology, Plant sciences

## Abstract

Plant-parasitic nematodes (PPNs) are vital soil organisms well-known to damage and reduce crop yield worldwide. Surveys were attempts to determine the impact of weed species on the communities and composition of nematodes in barley, wheat, quinoa, eggplant, and tomato crops in Alexandria and Ismailia regions of Egypt. During the surveys, eight occurring genera of nematodes were found namely; *Meloidogyne* spp, *Pratylenchus* spp, *Helicotylenchus* spp, *Rotylenchulus* spp, *Xiphinema* spp*, Criconemoides* spp, *Ditylenchus* spp*,* and *Longidorus* spp associated with the soil’s rhizosphere of 28 weed species belonging to 12 families. Among these weeds, *Hordeum marinum* and *Sonchus oleraceus* were good hosts to nematode species. Both wheat and barley had higher nematode diversity than quinoa in the winter season. *Pratylenchus* spp*, Meloidogyne* spp and *Rotylenchulus* spp can be considered vital potential PPNs with economic importance. Nematode abundances and structural indices varied greatly based on the host weed species, crop types and soil characteristics. A positive correlation was monitored among weeds, nematode frequencies and relative abundances as well as their crops. Finally, weed species are critical components in nematode communities that may increase the incidence and severity of nematode risks based on crop type and soil characteristics. Therefore weeds should be managed properly to diminish reservoir sites when developing nematode management options.

## Introduction

Plant parasitic nematodes have received much attention owing to the devastating disease and damage that they can cause^1,2^. Soil nematode communities of different lands could be used for comparison of soil health conditions in natural and managed ecosystems^3^. The plant-parasitic nematodes comprise the largest animal phylum in terms of the number of individuals and arguably the number of species^4^. One problem in determining the extent of crop loss due to plant parasitic nematodes is that the nematodes present are unknown in many areas^5^. These sophisticated soil-dwelling pests parasitize plant roots, which can alter the uptake of water and nutrients, interfere with the translocation of photosynthates^6^, and increase the incidence and severity of fungal wilt diseases^7,8^. Plant parasitic nematodes not only cause damage individually but form disease complexes with other micro-organisms and increase crop loss. Also, the symptoms of nematode damage are not specific and resemble the symptoms of other pathogens and abiotic stresses such as water and mineral deficiency^9^. These nematodes can cause severe root damage and some are capable of virus transmission^10^. Nematodes can be a limiting factor in field and vegetable crops^11^. *Meloidogyne incognita* is one of the most damaging nematodes of many crops^12^. Numerous plant-parasitic nematodes have a diverse spectrum of hosts while a few are host-specific. Overall, plant parasitic nematodes caused a projected yield loss of 12.3% worldwide annually; the losses are more in developing countries (14.6%) than in developed nations (8.8%). Based on this comprehensive survey on a global scale, the annual economic crop yield losses due to plant-parasitic nematodes in major crops have been estimated to be USD 173 billion^13^.

Weeds are known to host several species of plant parasitic nematodes^14^. The role of weeds as hosts for the plant parasitic nematode survival, development, reproduction, spread within a field, and increasing the potential for succeeding crops to be damaged by the nematode populations^15^. The significance of weeds acting as reservoir hosts is being increasingly recognized^16^. Weeds can act as reservoir hosts of a range of pests and diseases^17^. Weeds can have other less obvious effects, such as serving as a reservoir for insects^18,19^, diseases^20–22^, and nematodes^23,24^. Although competition is the most important effect of weeds on crop production and they have long been recognized for their ability to maintain nematode populations^23,25^. In the absence of a suitable crop host, weeds can act as alternative hosts for plant-parasitic nematodes and help to maintain nematode populations targeted for suppression by various management strategies^26^. Weeds not only serve as hosts to nematodes but they are also known to interfere with nematode management by protecting them from pesticides and adverse environmental conditions^26,27^. The interaction of weeds and nematodes can negatively impact crop production by reducing the nematode-suppressive benefits of crop rotations^25,28^. *Meloidogyne incognita* was reported to reproduce on the largest number of weeds with over 138 weedy plant hosts throughout the world^16^. In Egypt, previous studies have shown the presence of about 54 genera and 160 species of phytoparasitic nematodes associated with many crops and weeds^5,29^.

Understanding the distribution of plant-parasitic nematodes can help to make better decisions when it comes to controlling. Nematodes are very abundant, diverse and ubiquitous in all soils^30^. The vertical distribution of nematodes in the soil profile is highly variable and may be influenced by many biotic and abiotic factors^31^. The host plant (their food source) is the most important factor affecting nematode distribution in the soil profile^32^. Differences in the frequency, distribution and species composition of plant-parasitic nematode assemblage from one location to another may be attributed to climatic conditions, cropping practices and other environmental factors^33^. The distribution of nematode species varies greatly while the economic impact of their genera differs^34^. Nematodes are one of the major groups of soil organisms that are influenced by the presence of weeds and herbicide use^35^. Among weed species, the family Chenopodiaceae and Euphorbiaceae were good hosts of *M. arenaria,* and the family Polygonaceae and Portulaceae were moderate hosts. The Compositae and Convolvulaceae and Graminae and Solanaceae families were poor hosts for *M. arenaria*^36^. *Meloidogyne* spp. recorded infecting different weeds such as Common lambsquarters (*Chenopodium murale* L.), Small bindweed (*Convolvulus arvensis*), Common purslane (*Portulaca oleracea* L.) and Solanum (*Solanum nigrum*). The frequency of occurrence of root-knot nematodes *Meloidogyne* spp. showed different values in the surveyed locations^37^.

Nematodes have a wide host range and cause much more damage annually compared to other pests. This damage is further complicated by the presence of weeds due to the possible interactions. However, limited investigation exists about the relationship between weed species and nematodes as well as how these weeds can affect nematodes community and ecological indices in different crops in newly cultivated areas. This will present the importance of weed control to move toward more economical and environmental methods for nematode management. We hypothesized that the nematode communities and abundance are affected by various plant species. Further, we expect that crop-rich weed communities to be an associated with increased number and diverse communities of nematodes. Therefore, these studies aimed to provide more specific details on the association of weeds to NNP and describe the abundance of PPN communities in the field of selected crops. Comprehensive surveys are implemented to determine the potential association of nematodes and weed species and what population abundance, they have significant differences in their community composition. This information might help boost the sustainability of wheat, barley, eggplant tomato and quinoa productivity at newly cultivated lands in Alexandria and Ismailia governorate Egypt.

## Materials and methods

### Collection of soil samples

A survey of plant-parasitic nematodes was conducted in fields of wheat (*Triticum aestivum*) and barley (*Hordeum vulgare*), in Borg El-alarb at Alexandria governorate and quinoa (*Chenopodium quinoa*), Eggplant (*Solanum melongena*) and tomato (*Solanum lycopersicum*) in Ismailia governorate (Table 1). Borg El-alarb at Alexandria governorate in north-western Egypt (30° 52′ 1 84″ N, 29° 28′ 6 87″ E) is characterized by long dry summer and short cool rainy winter, typical of the Mediterranean climate. The mean winter temperature is 15°, the mean summer temperature is 26.0°. The total annual rainfall varies between 100 and 250 mm, confined to the months from November to March, with irregularities in quantity and distribution. Ismailia governorate located in north-eastern Egypt (30° 40′ 7 52″ N, 32° 29′ 5 46″ E) is characterized by a long dry summer and short cool rainy winter. The total annual rainfall varies between 61 mm, The average annual temperature is 27 °C, annual evaporation is 7.3/day, Wind N at 15–30 km/h, and the mean annual relative humidity is 66.3%.

**Table 1:** Soil chemical and physical analysis.

	Soluble anions (meq/l)				Soluble cations (meq/l)				EC dS/m	pH	Clay	Silt	Sand	Texture
	SO_4_^−−^	CL^−^	HCO_3_^−^	CO_3_^−−^	Mg^++^	Ca^++^	K^+^	Na^+^						
Brog El-arab	0.934	1.527	1.450	–	0.538	0.900	0.869	1.563	0.637	7.86	18.10	22.10	59.80	Sandy loam
Ismailia	2.250	3.620	1.430		2.243	2.720	0.520	1.850	1.027	8.02	16.00	19.00	65.00	Sandy loam

Samples were collected during three years (2020/2022) growing seasons in winter (wheat, quinoa and barely) planted in November and in summer (Eggplant & tomato) and planted in May and grown in monoculture. A total of 250 soil samples were randomly collected each year from the surveyed crops cultivated in newly reclaimed lands, these samples were taken at a depth of 0–20 cm from the rhizosphere of some plants. Sampling ensured that no nematicides were used during the study only conventional tillage and agronomic practices were used during the growing seasons. The samples were collected in N shaped pattern across each field and year. Soil samples were kept in a polyethylene bag, and sent directly to the laboratory for nematode extraction and analysis.

### Laboratory for nematode extraction and identification

The soil sample of each plant was carefully mixed with five replicates from each field twice during the season before the fruiting stage and once before harvesting. After being collected, soil samples were taken to the Nematology lab at the Desert Research Center and kept at 4 °C until processing. Field history data, such as crop rotation practices, fallow times, tillage practices, and pest management are obtained from the grower at the time of sampling to detect the possibility of connections between nematodes and management strategy.100 g soil was added to a glass beaker (1000 ml) and the mixture was agitated for a few seconds, then, an aliquot of soil suspension was processed for nematode extraction using a set of sieves from 60 to 350 mesh. After that nematodes were collected from the suspension using rapid flotation-sieving technique, and concentrated to 50 ml in vials using 350 mesh sieves. The nematodes were counted in 1 ml using a Hawkesly counting slide and identified to the generic level under a stereo microscope decanting method^38^.

### Nematode identification

The surveyed nematodes were identified at the generic level using morphological traits of adult and larval forms such as using overall body shape size, stylet shape, esophagus and intestine orientation, amphid opening, tail shape, metacorpus size, vulva position and several other measures of gross body morphology^39–41^.

### Weed demographic analysis

The survey was conducted according to^42^ protocols randomly to avoid anomalies in the fields. Weed specimens were collected from each field twice during the season before the fruiting stage and once before harvesting at the same time with nematode sampling and identified by plant taxonomy specialists at the Desert Research Center^43^. Sampling ensured that no herbicides were implemented in the growing season and earlier to manifest an effect on weed populations. Five sampling areas of hundred square meters in the middle and four directions with three replications were taken in a W-pattern across each field to investigate the weed community and its dominated nematodes and to count individuals of each species from each quadrate. Density measures per 100 m^2^ are expressed as a percent of the total number of a single weed species. Percent frequency was calculated from the percentage of number samples that were positive (with associated nematodes) for each weed per 100 square meters. The qualitative and quantitative analysis of vegetation differences was implemented to obtain frequency percentages and relative abundances under each location’s environmental conditions.

### Data analysis

Under a stereoscopic microscope, the number of nematodes of each genus in a counting dish was counted to assess the nematode population. Then, abundance was expressed as the number of nematodes per 100 g soil and then used to determine the relative abundance that was calculated as the number of positive samples/ number of collected soil samples × 100. Percent frequency was calculated from the percentage of samples that were positive (associated with nematodes) of a certain nematode genus as the total number of samples in which that genus was detected, divided by the total number of samples collected and multiplied by 100. The frequencies of occurrence and relative abundance of nematodes and weed species have been correlated using Pearson correlation coefficient using the computer program Sigmaplot 12.5.

## Results

The frequencies of occurrence % (F.O. %) and relative abundances % (R. Ab. %) of nematode coexisting with the rhizosphere soil of several weeds were recorded in five cultivated fields. Whereas the soil levels of EC (0.637 and 1.027%), and pH (7.86 and 8.02) were higher in Ismailia than in Borg El arab region respectively as well as all soluble anion and cations with the same sandy loam soil textures (Table 1).

The survey revealed that eight different nematode genera were included *Meloidogyne* spp*, Rotylenchulus* spp*, Pratylenchus* spp*, Ditylenchus* spp*, Criconemoides* spp*, Helicotylenchus* spp*, Xiphinema* spp, and *Longidorus* spp that harbored by 28 weeds. They encountered 13 annual winter weeds represented 46.43%, 11 annual summer weeds represented 39.29% and perennial weeds represented 14.29% of the total weeds numbers belonging to 12 families. The total nematode percentage reached 56.20% of winter annual weeds, 28.93% of summer annuals, and 14.88% of perennial weeds. On the other hand, the most prevalent genera of nematodes was *Pratylenchus* spp (21.7%), followed by *Meloidogyne* spp (19.1%), then *Rotylenchulus* spp (17.4%), *Helicotylenchus* spp (15.7%) respectively, however, the lowest genera was *Longidoru*s spp (2%) in the soil rhizosphere of weeds (Table 2).

**Table 2:** Host plants of nematodes genera and their percentage in weeds.

Numbers	Type	Family	Weeds	Meloidogyne	Pratylenchus	Helicotylenchus	Rotylenchulus	Xiphinema	Ditylenchus	Criconemoides	Longidorus	%
1	Annual winter	Poaceae	*Avena fatua*	*	*	*		*		*		68.0
2		Poaceae	*Avena sativa*	*	*		*	*	*			
3		Poaceae	*Lolium multiflorum*		*		*	*	*	*		
4		Poaceae	*Hordeum marinum*	*	*	*	*	*	*	*		
5		Poaceae	*Polypogon monspeliensis*	*	*		*	*	*		*	
6		**Asteraceae**	*Sonchus oleraceus*	*	*	*	*	*	*		*	
7		**Asteraceae**	*Silybum marianum*	*	*	*	*					
8		**Fabaceae**	*Melilotus* indicus	*	*	*	*	*		*		
9		**Polygonaceae**	*Rumex dentatus*		*			*			*	
`10		**Amaranthaceae**	*Beta vulgaris*		*		*					
11		Amaranthaceae	*Chenopodium album*	*	*	*	*	*				
12		**Malvaceae**	*Malva parviflora*	*	*	*	*	*	*			
13		**Brassicaceae**	*Raphanus raphanism*	*	*		*			*		
14	Annual summer	Poaceae	*Echinochloa colonum*	*	*	*	*					35.0
15		Poaceae	*Echinochloa crus-galli*	*	*		*					
16		**Portulacaceae**	*Portulaca oleracea*	*	*	*	*					
17		**Asteraceae**	*Conyza canadensis*	*		*						
18		**Asteraceae**	*Xanthium strumarium*	*	*							
19		**Euphorbiaceae**	*Euphorbia peplus*	*	*							
20		**Malvaceae**	*Hibiscus trionum*	*	*	*	*					
21		**Malvaceae**	*Corchorus olitorius*	*		*	*					
22		**Malvaceae**	*Sida alba*	*	*	*	*					
23		**Amaranthaceae**	*Amaranthus retroflexus*	*	*	*						
24		Solanaceae	*Solanum nigrum*	*	*	*	*					
25	Perennial weeds	**Convolvulaceae**	*Convolvulus arvensis*	*	*	*	*	*				18.0
26		Poaceae	*Imperata cylindrica*		*			*		*		
27		Poaceae	*Cynodon dactylon*	*	*	*	*		*	*		
28		**Solanaceae**	*Solanum elaeagnifolium*		*	*	*		*			
		**%**		19.1	21.7	15.7	17.4	10.4	7.0	6.1	2.6	

*The presence of nematodes associated with rhizosphere soil.

In wheat fields, six genera (PPN) namely; *Meloidogyne* spp, *Rotylenchulus* spp, *Pratylenchus* spp, *Ditylenchus* spp, *Criconemoides* spp, and *Helicotylenchus* spp were prevalently detected in the soil rhizosphere of weeds (Table 3). The rich weed hosts were *M. parviflora* which coexist with six genera of nematodes followed by *M. indicus, H. murinum* and *C. arvensis* associated with five genera of plant nematode, on the other hand, humble hosts were *P. monspeliensis*, *I. cylindrica* and *C. album* associated with two genera of nematode. The total frequencies of the nematode ranged from 23.64 to 10.91% of the total samples in wheat fields. The highest weed associated with nematode was *M. parviflora* reaching 10.91*%* (frequencies) and 10.77% (relative abundance) respectively. Fifteen weed species belonging to 9 families were associated with nematodes in their rhizosphere soil. The frequencies of weeds ranged from 14.85 to 0.90% of the total samples. The highest weed frequency was *L. temulentum* (14.85%)*,* followed by *H. murinum* (13.50%) respectively. While, *A. fatua (*15.96%) was recorded as the highest nematode abundance, followed by *A. sativa* (14.89%) respectively. Pearson correlation analysis was strongly positive (0.177) of frequencies between nematodes & weeds. However, the person correlation was strongly negative (-0.35) in wheat fields.

**Table 3:** Frequency of occurrence and population relative abundance of weeds and Nematodes found in rhizospheric soil of weeds in wheat fields.

Nematodes	Nematodes types (100 g rhizospheric soil)		Associated weeds*	Total nematode distribution (100 g rhizospheric soil)		Weed distribution (100 m^2^)	
	F.O %	R. Ab. %		F.O %	R. Ab. %	F.O %	R. Ab. %
*Meloidogyne* spp^1^	18.18	27.27	*A. fatua*^1,3,6^	5.45	5.53	12.15	15.96
*Rotylenchulus* spp^2^	23.64	24.95	*A. sativa*^1,2,3^	5.45	4.87	5.40	14.89
*Pratylenchus* spp^3^	21.82	23.81	*H. murinum*^2,3,4,5,6^	9.09	9.44	13.50	11.70
*Ditylenchus* spp^4^	10.91	12.09	*L. temulentum*^2,4,5^	5.45	2.99	14.85	11.49
*Criconemoides* spp^5^	14.55	5.81	*P. monspeliensis*^2,3^	3.64	3.42	8.26	12.55
*Helicotylenchus* spp^6^	10.91	6.07	*S. oleraceus*^1,2,3,4^	7.27	6.49	1.35	4.47
			*M. indicus*^1,2,3,5,6^	9.09	10.89	9.85	4.89
			*M. parviflora*^1,2,3,4,5,6^	10.91	10.77	13.50	3.19
			*C. album*^1,2^	3.64	3.20	6.75	7.66
			*S. mariannm*^1,2,5^	5.45	4.82	3.60	2.55
			*R. raphanistrum*^1,2,3,5^	7.27	6.49	0.90	2.34
			*B. vulgaris*^1,2,3,5^	7.27	6.42	0.90	1.06
			*C. arvensis*^1,2,3,4,6^	9.09	14.53	2.25	1.70
			*I. cylindrica*^3,5^	3.64	2.65	2.70	2.55
			*S. elaeagnifolium*^2,3,4,6^	7.27	7.48	4.05	2.98
Mean SD (standard deviation) between 3 years	± 5.6	± 6.4		± 6.2	± 3.4	± 4.5	± 3.2

Correlation between frequencies = 0.168. Correlation between abundances = −0.35.

Regression r^2^ between frequencies = 0.028. Regression r^2^ between abundances = 0.028.

*SD* standard deviation between the 3 years.

*Number of collected soil samples 50 1, 2, 3… = refers to nematodes genera.

Similar to wheat, there were six genera of *Meloidogyne* spp*, Rotylenchulus* spp*, Pratylenchus* spp*, Ditylenchus* spp*, Criconemoides* spp*, Helicotylenchus* spp coexist of weed rhizosphere soil in barely fields (Table 4). Their respective ranging of frequencies and relative abundance were 26.42 to 9.43% and 26.22 to 9.25%, respectively. The rich hosts were *M. indicus, S. oleraceus*, *A. fatu* and *S. mariannm* coexist with 4 plant parasitic nematodes genera*,* however, the poor weed host was *X. stramonium* associated with two plant parasitic nematodes. The highest frequency was recorded from *M. indicus* (9.26%) while, *S. mariannm* has the highest nematode abundance (12.31%), followed by *S. elaeagnifolium* (9.72%) of total nematodes. In terms of weed occurrence, nematode was recorded predominately in the rhizosphere soil of 15 weed species belonging to nine families. They have frequencies ranging from 8.50 to 3.27% of the samples. The most frequent weed was *M. parviflora* occurred in barley fields. *A. fatua* was the highest abundance weed by 15.96%, followed by *A. sativa.* Between nematodes & weeds frequencies, Pearson correlation analysis was strongly positive (0. 224). However, the correlation was strongly negative (− 0.440) in barley fields.

**Table 4:** Frequency of occurrence and population relative abundance of weeds and nematodes found in rhizospheric soil of weeds in barley fields.

Nematodes	Nematodes types (100 g rhizospheric soil)		Associated weeds	Total nematode distribution (100 g rhizospheric soil)		Weed distribution (100 m^2^)	
	F.O %	R. Ab. %		F.O %	R. Ab. %	F.O %	R. Ab. %
*Meloidogyne* spp^1^	20.75	26.22	*A. fatua*^1,2,3,5^	7.41	3.78	7.84	15.96
*Rotylenchulus* spp^2^	26.42	21.01	*A. sativa*^1,2,4^	5.56	5.83	7.19	14.89
*Pratylenchus* spp^3^	20.75	18.53	*H. murinum*^1,2,3^	5.56	5.83	6.54	11.70
*Ditylenchus* spp^4^	13.21	15.07	*L. temulentum*^2,3,4^	5.56	5.51	7.84	11.49
*Criconemoides* spp^5^	9.43	9.91	*P. monspeliensis*^1,2,4^	5.56	4.32	7.19	12.55
*Helicotylenchus* spp^6^	9.43	9.26	*S. oleraceus*^1,2,3,4^	7.41	4.78	5.88	4.47
			*M. indicus*^1,2,3,5,6^	9.26	7.18	7.84	4.89
			*M. parviflora*^1,2,3,6^	7.41	7.56	8.50	3.19
			*C. album*^1,2,4,5^	7.41	9.18	7.84	7.66
			*S. mariannm*^1,2,3,6^	7.41	12.31	3.27	2.55
			*R. raphanistrum*^1,2,5^	5.56	4.86	3.92	2.34
			*B. vulgaris*^1,2^	3.70	4.59	5.88	1.06
			*C. arvensis*^1,2,3,6^	7.41	8.10	7.19	1.70
			*I. cylindrica*^2,3,4,6^	7.41	6.48	6.54	2.55
			*S. elaeagnifolium*^2,3,4,6^	7.41	9.72	6.54	2.98
Mean SD between 3 years	± 4.5	± 6.5		± 1.2	± 2.6	± 1.5	± 5.2

Correlation between frequencies = 0.224. Correlation between abundances = −0.440.

Regression r^2^ between frequencies = 0.050. Regression R^2^between abundances = 0.050.

The presence of five PPN genera of *Meloidogyne* spp, *Rotylenchulus* spp, *Xiphinema* spp, *Pratylenchus* spp*,* and *Longidorus* spp was the most prevalent found in the quinoa fields (Table 5). They have frequencies ranging from 35.14 to 10.81% and relative abundance ranging from 27.77 to 9.61% of the samples, respectively. The good host was *B. vulgaris* associated with the five nematode genera*,* followed by *Rumex dentatus* and *Sonehus oleraceus* hosted 4 nematode genera. The highest frequencies were found in *B. vulgaris* reaching 13.51% of total nematodes. While, the highest nematode relative abundance was *C. arvensis* (14.95%) followed by *B. vulgaris* (12.51%)*,* respectively*.* However, the lowest abundance was found by 2.93% associated with *I. cylindrica*. The occurrence of nematodes coexisting in the rhizosphere soil of 13 weed species belonging to 7 families was recorded. The weed frequencies ranged from 23.47 to 4.08% of the collected samples. *C. album* was found to have the highest frequency. The highest weed relative abundance was *H. murinum* by 14.74%*.* The correlation between frequencies of nematodes & weeds was strongly positive (0.730). However, the correlation between nematodes and weeds was strongly negative (-0.389) in quinoa fields.

**Table 5:** Frequency of occurrence and population relative abundance of weeds and nematodes found in rhizospheric soil of weeds in quinoa fields.

Nematodes	Nematodes types (100 g rhizospheric soil)		Associated weeds	Total nematode distribution (100 g rhizospheric soil)		Weed distribution (100 m^2^)	
	F.O%	R. Ab. %		F.O%	R. Ab. %	F.O%	R. Ab. %
*Meloidogyne* spp^1^	24.32	9.61	*A. fatua*^1,2,3^	8.11	7.13	7.14	10.90
*Rotylenchulus* spp^2^	10.81	24.69	*A. sativa*^1,3^	5.41	5.63	5.10	7.69
*Xiphinema* spp^3^	35.14	27.77	*H. murinum*^2,3^	5.41	5.23	4.08	14.74
*Pratylenchus* spp^4^	18.92	18.56	*L. temulentum*^3,4^	5.41	5.01	5.10	10.26
*Longidorus* spp^5^	10.81	19.37	*P. monspeliensis*^3,5^	5.41	5.85	5.10	12.18
			*S. oleraceus*^1,2,3,4^	10.81	8.45	6.12	1.92
			*M. indicus*^3^	5.41	2.99	5.10	2.56
			*C. album*^1,3,4^	8.11	11.44	23.47	12.18
			*M. parviflora*^1,3,4^	8.11	5.81	7.14	14.10
			*B. vulgaris*^1,2,3,4,5^	13.51	12.71	10.20	1.28
			*R. dentatus*^1,3,4,5^	10.81	11.88	8.16	1.92
			*C. arvensis*^1,2,3^	8.11	14.95	8.16	3.21
			*I. cylindrica*^2,3^	5.41	2.93	5.10	7.05
Mean SD between 3 years	± 6.7	± 8.2		± 4.7	± 3.6	± 3.1	± 4.9

Correlation between frequencies = 0.730. Correlation between abundances = −0.389.

Regression r^2^ between frequencies = 0.533. Regression R^2^between abundances = 0.086.

In eggplant fields, *Meloidogyne* spp*, Rotylenchulus* spp, *Pratylenchus* spp, and *Helicotylenchus* spp, are present to coexist with weed rhizosphere soil (Table 6). Whereas, most weeds were harbored by *Meloidogyne* spp followed by *Pratylenchus* spp and *Helicotylenchus* spp respectively of weeds. *M. indicus*, *P. monspelensis*, *M. parviflora* and *A. fatua* were good hosts that coexist with four nematodes genera while *I. cylindrica* was a poor host coexisting with one nematode genera. Their relative frequencies and abundances ranged from 34.88 to 18.60% and 42.11 to 10.88%, respectively. The highest frequency of nematode was found in coexistence with *E. colonum*, *P. oleracea*, *H. trionum* and *S. alba* reaching 10.00*%*. While, the highest abundance was *H. trionum* (12.45%), followed by *E. colonum* (11.64%) of nematodes. The occurrence of nematodes coexists in 13 weed species rhizosphere soil belonging to 8 families was recorded in eggplant fields. The weeds, frequencies ranged from 16.22 to 2.70%, while, *E. crus-galli* achieved the largest frequency and abundance of 16.22 and 14.11% respectively. The correlation between nematodes and weeds was strongly positive (0. 107) of the frequencies. However, between nematodes and weeds, the correlation was strongly negative (−0.153) in eggplant fields.

**Table 6:** Frequency of occurrence and population relative abundance of weeds and nematodes found in rhizospheric soil of weeds in eggplant fields.

Nematodes	Nematodes types (100 g rhizospheric soil)		Associated weeds	Total nematode distribution (100 g rhizospheric soil)		Weed distribution (100 m^2^)	
	F.O%	R. Ab. %		F.O%	R. Ab. %	F.O%	R. Ab. %
*Meloidogyne* spp^1^	34.88	42.11	*E. crus-galli*^1,2^	5.00	5.19	16.22	14.11
*Rotylenchulus* spp^2^	20.93	22.62	*E. colonum*^1,2,3,4^	10.0	11.64	10.81	11.69
*Pratylenchus* spp^3^	25.58	24.39	*S. nigrum*^1,2,3^	7.50	5.85	8.11	10.08
*Helicotylenchus* spp^4^	18.60	10.88	*C. olitorius*^1,2,4^	7.50	6.79	8.11	4.84
			*P. oleracea*^1,2,3,4^	10.00	5.35	10.81	8.06
			*X. strumarium*^1,2,3^	7.50	6.29	5.41	2.42
			*E. peplus*^1,4^	7.50	2.14	2.70	1.61
			*H. trionum*^1,4^	10.00	12.45	5.41	4.44
			*S. alba*^1,2,3,4^	10.00	10.47	13.51	10.89
			*C. canadens*^1,4^	5.00	7.04	5.41	4.44
			*A. retroflexus*^1,3^	5.00	4.78	5.41	10.89
			*C. dactylon*^1,2,3,4^	7.50	11.32	2.70	9.68
			*C. arvensis*^1,2,3^	7.50	10.69	5.41	6.85
Mean SD between 3 years	± 3.6	± 4.8		± 1.8	± 3.4	± 4.0	± 3.2

Correlation between frequencies = 0.107, correlation between abundances = 0.153.

Regression r^2^ between frequencies = 0.011, regression R^2^between abundances = 0.023.

Table 7 indicated four PPN genera of *Meloidogyne* spp, *Rotylenchulus* spp, *Helicotylenchus* spp*,* and *Pratylenchus* spp were associated with weeds in tomato fields. All weeds were good hosts to coexist with *Meloidogyne* spp and *Pratylenchus* spp. Both occurrences of frequencies and relative abundances ranged from 36.67 to 13.33% and 40.69% to 10.15%, respectively. The highest frequency was found in *S. nigrum* by 13.33% of the total nematodes. While, *C. arvensis* and *E*. *crus-galli* had the highest nematode abundance by 13.96 and 13.65%, respectively. The occurrence of nematodes coexisting in the rhizosphere soil of 11weed species belonging to 8 families was recorded. The highest weed frequency and abundance were *E. crus-galli* reaching 15.69 and 15.92% respectively. The correlation analysis was strongly positive (0.177) between the frequencies of nematodes & weeds. However, the correlation analysis was strongly negative (− 0.360) between the abundances of nematodes and weeds in tomato fields.

**Table 7:** Frequency of occurrence and population relative abundance of weeds and Nematodes found in rhizospheric soil of weeds in tomato fields.

Nematodes	Nematodes types (100 g rhizospheric soil)		Assoc. weeds	Total Nematode distribution (100 g rhizospheric soil)		Weed distribution (100 m^2^)	
	F.O %	R. Ab. %		F.O%	R. Ab. %	F.O%	R. Ab. %
*Meloidogyne *spp^1^	36.67	40.69	*E. crus-galli*^1,4^	6.67	13.65	15.69	15.92
*Rotylenchulus* spp^2^	13.33	24.99	*E. colonum*^1,2,3^	10.00	6.23	9.80	11.46
*Helicotylenchus* spp^3^	23.33	24.17	*S. nigrum*^1,2,3,4^	13.33	14.94	9.80	19.11
*Pratylenchus* spp^4^	26.67	10.15	*C. olitorius*^1,4^	6.67	6.42	7.84	6.37
			*P. oleracea*^1,2,3,4^	10.00	7.02	11.76	6.37
			*X. strumarium*^1,4^	6.67	5.74	5.88	5.10
			*E. peplus*^1,4^	6.67	12.57	5.88	2.55
			*H. trionum*^1,3,4^	10.00	8.45	7.84	11.46
			*A. retroflexus*^1,3,4^	10.00	8.15	9.80	10.19
			*C. dactylon*^1,3,4^	10.00	2.87	11.76	7.01
			*C. arvensis*^1,2,3^	10.00	13.96	3.92	4.46
Mean SD between 3 years	± 5.6	± 6.4		± 2.2	± 3.4	± 3.5	± 4.8

Correlation between frequencies = 0.177, correlation between abundances = 0.360.

Regression r^2^ between frequencies = 0.031, regression R^2^ between abundances = 0.130.

## Discussion

Bio-aggressors magnitude the impact of nematodes in productivity loss of agriculture fields at newly reclaimed lands of Egypt. Furthermore, there was plenty of scientific proof that weeds may promote the establishment of plant-parasitic nematodes and duplicate their risks to crop production. However, a few studies were made to determine the relationship between weeds and plant-parasitic nematodes. Plant parasitic nematodes are obligate parasites that some crops are not preferred hosts so their population will decline over time if not found other hosts. In the absence of host crops, the seeds serve as transitional hosts for the population’s survival which may contribute to the maintenance, earlier infestation, multiplication, and spread of plant parasitic nematodes within a field, and thus an increase in the potential for crops to be damaged by nematode attacks^23,44^. Weeds not only act as alternative hosts for plant-parasitic nematodes but also the presence of weed hosts reduces the efficacy of nematode management strategies^25,26^.

This study proved the associations of several genera of plant-parasitic nematodes with weeds via direct relationship to cause major threats to agriculture production. These extensively include monocotyledonous and dicotyledonous weeds and showed that nematodes can live on a wider range of host plants. The weed genotypes can be classified as susceptible (good host) or resistant (poor host) to nematodes depending on the size of their reproduction. The performance of each category will vary with the status of the crop to a specific nematode^45^. Nematode-susceptible weeds have the possibility of maintaining high nematode population levels and will be tremendously damaged. If so then the weed-infested fields have the potential to reduce the effectiveness of nematode-resistant crops^46,47^. Therefore, it could minimize the nematode damage to crops through the use of nematicides, and plant resistance, or minimize the weed infestation by herbicides, to preserve the ultimate ability of the crop to be as competitive as possible with weeds. Weeds also serve as a reservoir, secondary host, or transport for diseases^22,48^.

To identify the occurring nematodes through this study, eight occurring genera with varying degrees of morphological have been discovered namely root-knot (*Meloidogyne*), root-lesion (*Pratylenchus*), spiral nematode (*Helicotylenchus*), reniform nematode (*Rotylenchulus*), dagger Nematode (*Xiphinema*), ring Nematodes (Criconemoides), stem nematode (Ditylenchus), and needle nematode (*Longidorus*). The most prevalent genera of nematodes was *Pratylenchus* with a percentage of 21.7%, followed by *Meloidogyne* with 19.1%, then *Rotylenchulus* with 17.4% and *Helicotylenchus* with 15.7% coexist in the soil rhizosphere of weeds. *Meloidogyne* spp is the most damaging one because of its ability to feed on a greater range of crop plants. *Meloidogyne* and *Rotylenchulus* represent the highest population abundances, while *Rotylenchulus* are widespread genera in the majority of investigated field crops*.* According to the findings, the predominant genera in the quinoa field were *Xiphinema*. *Meloidogyne* was the most prevalent genus of plant-parasitic nematodes identified in tomato fields. Another significant genus was *Pratylenchus* (root-lesion) which is one of the most economically damaging lesion nematodes found in eggplant fields and a wide variety of crops. One of the most important migratory endoparasitic nematodes that affect corn is the lesion nematode (*Pratylenchus spp*)^49^. The second potential predominant genera were *Ditylenchus* spp*, Criconemoides* spp and *Helicotylenchus* spp. however, the lowest prevalent genera were *Longidorus spp* needle nematode in the tested crops. Therefore, we can fairly conclude that plant-parasitic nematodes are quite common in the selected fields in the two regions based on these results. By comparing the presence of nematodes on winter weeds of wheat and barley in the Borg Al Arab region and in quinoa in the Ismailia region. The occurrence was affected by the nature of the soil and decreased in presence by increasing salinity via the level of soluble anions and cations. Soil physical characteristics can affect the occurrence and population dynamics of nematodes^50^. Therefore, we quantified the relationships of weeds, and plant-parasitic nematodes, which appeared a similar variability in their community composition related to the studied years and fields. However, the presence and abundance of nematodes strongly may differ from one and another weed species. We found that most studied plant-parasitic nematodes have a wide host range of weeds as alternative hosts. The most common host preferences are *H. marinum* and *S. oleraceus,* which widely serve as reservoirs of nematodes*.* While *L.multiflorum, P. monspeliensis* (annul narrow leaved weeds)*, M. indicus, M. parviflora, R. raphanism* (broadleaved weeds) and *C. dactylon* (perennial weeds) were moderate hosts to nematodes*.* Based on the person correlation the increase in weed frequencies is always accompanied by a rise in the nematode frequencies. While relative abundance refers to a higher reservoir capacity of weeds to contain a high nematode population. A positive correlation was monitored by 0.846 (frequencies of occurrence) and 0.685 (abundances) between crops and nematodes, while, A positive correlation was monitored by 0.668 (frequencies of occurrence) and 0.761 (abundances) between crops and weeds respectively. It could be concluded that nematode assemblage differences may be attributed to vegetation cover of weeds and crops as well as other environmental factors including soil characteristics. Accordingly, weeds were highly preferred hosts in nematode populations than crops and may played an important role in their growth and distribution. The abundance of weeds in a given field and the rate of nematode reproduction on specific weed species determine the magnitude of the effect that they may have on the build-up of plant-parasitic nematode population densities^51^. This was in line with our assumption that rich fields with weed plants have more PPN populations with meaningful frequencies and abundances in the nematode communities. Therefore, the higher diversity and number of nematodes associated with wheat crops are due to their diverse weed species. This knowledge will help us to better understand and manage factors affecting nematodes' presence and weeds and their impacts on the succeeding crops. Therefore, applying crop rotation is a necessity for controlling weeds, particularly during idle periods between crops that allow prolific weed growth and benefit nematode management. Also, this study on nematode presence and variability will be valuable to the establishment of future studies about the damage of each plant-parasitic nematode genera under Egypt conditions and to the development of nematode management options.

## Data Availability

The datasets used during the current study are available from the corresponding author upon reasonable request.
